# Collinear Stimuli Induce Local and Cross-Areal Coherence in the Visual Cortex of Behaving Monkeys

**DOI:** 10.1371/journal.pone.0049391

**Published:** 2012-11-19

**Authors:** Ariel Gilad, Elhanan Meirovithz, Amir Leshem, Amos Arieli, Hamutal Slovin

**Affiliations:** 1 The Gonda Multidisciplinary Brain Research Center, Bar-Ilan University, Ramat Gan, Israel; 2 School of Engineering, Bar Ilan University, Ramat-Gan, Israel; 3 Weizmann Institute of Science, Neurobiology Department, Rehovot, Israel; University College London, United Kingdom

## Abstract

**Background:**

Collinear patterns of local visual stimuli are used to study contextual effects in the visual system. Previous studies have shown that proximal collinear flankers, unlike orthogonal, can enhance the detection of a low contrast central element. However, the direct neural interactions between cortical populations processing the individual flanker elements and the central element are largely unknown.

**Methodology/Principal Findings:**

Using voltage-sensitive dye imaging (VSDI) we imaged neural population responses in V1 and V2 areas in fixating monkeys while they were presented with collinear or orthogonal arrays of Gabor patches. We then studied the spatio-temporal interactions between neuronal populations processing individual Gabor patches in the two conditions. Time-frequency analysis of the stimulus-evoked VSDI signal showed power increase mainly in low frequencies, i.e., the alpha band (α; 7–14 Hz). Power in the α-band was more discriminative at a single trial level than other neuronal population measures. Importantly, the collinear condition showed an increased intra-areal (V1-V1 and V2-V2) and inter-areal (V1-V2) α-coherence with shorter latencies than the orthogonal condition, both before and after the removal of the stimulus contribution. α-coherence appeared between discrete neural populations processing the individual Gabor patches: the central element and the flankers.

**Conclusions/Significance:**

Our findings suggest that collinear effects are mediated by synchronization in a distributed network of proximal and distant neuronal populations within and across V1 and V2.

## Introduction

Contextual effects in the visual system have been studied using collinear configurations of local elements as well as using contour integration. The latter occurs when proximal discrete elements with similar orientation are grouped together to generate a continuous and salient contour that “pops out” from its background [1]–[4]. Contour integration follows the Gestalt law of “good continuation” [5] and the smallest fragment of a contour that obeys this law is a collinear element.

Psychophysical studies reported that adding proximal collinear flankers to a central element (i.e. the ‘target’), can reduce the contrast threshold detection of this ‘target’ [6]–[15]. This effect is reduced when the flankers’ orientation is orthogonal to the target’s orientation [8], [10], suggesting that facilitation in ‘target’ detection is related to “good continuation”. Anatomical studies suggest that the long horizontal connections in the primary visual cortex (V1) play a major role in collinear influences and contour integration [16]–[21].

How can discrete neuronal assemblies in the visual cortex, each activated by an individual visual element (i.e. a Gabor element or a line segment) “interact” to mediate contextual effects? Previous electro-physiological studies [7], [8], [22] have shown that collinear elements can facilitate (but see [14]) the neuron’s firing rate to a low-contrast ‘target’ presented in the receptive field (we shall refer to the ‘target’ as the central element, CE). While these studies and others [23]–[26] have demonstrated mainly rate modulation, other reports have suggested synchrony to play an important role in mediating contextual effects [27]–[30]. Despite extensive research efforts [25], [31]–[37], the role of neural synchronization, particularly between different visual areas, e.g. V1 and V2, in contextual effects is not well understood. Here, we focus on studying the intra- and inter-areal interactions between neuronal populations processing individual Gabor elements arranged in a collinear or orthogonal configuration.

In a recent study [28], we found that an increased correlation (denoted as ‘onset synchronization’) among neuronal populations processing the CE in V1 was higher for collinear than for orthogonal stimulus. However, it was not clear whether this correlation extend beyond V1 to a larger network. In particular, the role of synchrony between V1-V2 areas and between V2-V2 areas in the collinear condition has not been studied in depth. Investigating these relations is sensitive to noise perturbations and therefore, we implemented a spectral approach and used a coherence analysis which enabled us to study the intra- and inter-areal synchrony in relation to distinct frequency bands of the VSDI signal. In addition, to investigate whether the intra-areal and inter-areal coherence involves internal cortical processing (rather than ‘onset synchronization’ that reflects mainly correlated neural activity directly evoked by stimulus), we studied the coherence after removal of visual stimulus contribution.

Here, we performed voltage-sensitive dye imaging (VSDI) at high spatial and temporal resolution in the visual cortex of fixating monkeys during stimulus presentation. As shown previously, we found that the spectral content of the stimulus-evoked VSDI signal was composed mainly of low frequencies, i.e. the alpha band (α; 7–14 Hz; [38]). Coherence in the α-band was computed between discrete neuronal populations in areas V1 and V2 processing individual Gabor patches of collinear or orthogonal arrays of Gabors, both before and after removing the stimulus contribution. In the collinear condition, the α-coherence among discrete and remote neuronal populations in V1 and V2 areas showed higher values and faster dynamics.

## Results

Our main goal was to study both intra-areal and inter-areal synchronization of neural populations and their relation to contextual effects of collinear and orthogonal stimuli. We therefore trained two monkeys on a fixation task while they were presented on separate trials with either a collinear or an orthogonal stimulus. Each stimulus was comprised from a central Gabor element (CE) embedded within collinear flankers (collinear stimulus) or within orthogonal flankers (orthogonal stimulus). We then measured the population responses from V1 and V2 areas using voltage-sensitive dye imaging (VSDI). VSDI time-frequency analysis is well suited to study intra-areal and inter-areal synchronization as it enables investigating the relations among distributed neural assemblies using specific frequency bands of the signal at high spatial and temporal resolution [39], [40]. Data were obtained from a total of 8 VSDI recording sessions from two hemispheres of two monkeys.

### Retinotopic Mapping of the Central Element and the Flanker Element in V1 and V2

**Figure 1:**
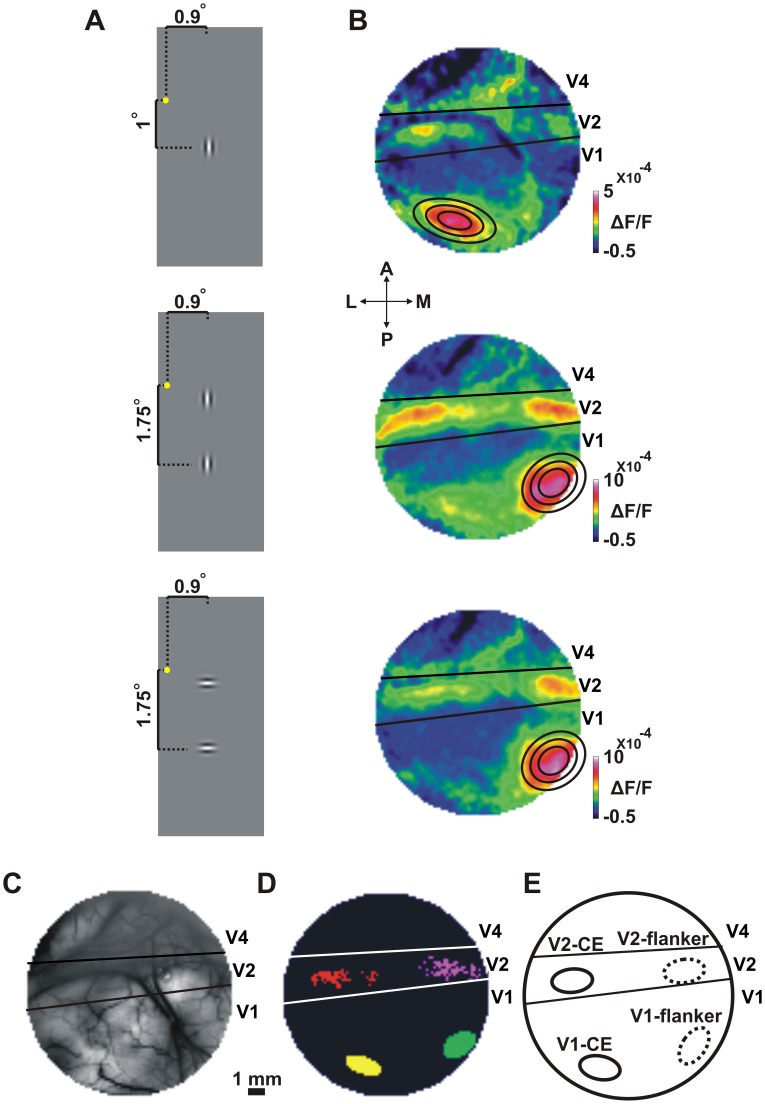
Retinotopic mapping of the central element and the flanker. *A*: Schematic illustration of the Gabor stimuli used for retinotopic mapping: the central element (CE, top) and the two flankers, vertically (middle) or horizontally (bottom) oriented were presented in separate trials. Fixation point is marked with a yellow dot. *B*: Average population response (VSDI amplitude) maps evoked by the stimuli in A. Maps and stimuli in A are presented in a corresponding order (from top to bottom). Color denotes fluorescence change (ΔF/F). The maps show activation patches in V1 for the central element (V1-CE activated area) and the lower flanker (V1-flanker activated area; the upper, more foveal flanker evoked neuronal activation outside the imaged area). In V1, a 2D Gaussian fit of the neuronal activation at the V1-CE activated area (top) and V1-flanker activated area (middle and bottom) are superimposed on the corresponding maps. Inner to outer contours represent the top 10%, 20% and 40% outlines of the Gaussian, respectively. Pixels within the top 10% fitted Gaussian area were defined as the ROIs in V1. In V2 we defined V2-CE and V2-flanker ROIs as pixels exceeding an SNR threshold. *C*: Blood vessel pattern of the imaged area. Black lines mark the border between V1/V2 and the lunate sulcus (LUS) located between V2 and V4. *D*: The four ROIs taken for analysis in this specific recording session: V1-CE (yellow), V1-flanker (green) and V2-CE (red) and V2-flanker (purple). *E:* A schematic illustration of the four ROIs in D in a more general outline. The schematic illustrations in E and D will be used throughout the following Figs.

Our first step was to map the individual Gabor elements comprising the collinear or orthogonal stimuli onto V1 and V2 areas. For this purpose, the monkeys were presented during fixation with the Gabor central element (CE) alone (Fig. 1A top) or the two flankers most proximal to the CE (Fig. 1A middle and bottom for vertically and horizontally oriented flankers respectively). Fig. 1B displays the time average population response maps evoked by these stimuli in V1 and V2. The response map in V1 shows a clear patch corresponding to the CE alone (Fig. 1B top) and another patch corresponding to the lower flanker (Fig. 1B, middle and bottom; the upper flanker was positioned at a more foveal location and therefore the evoked neuronal activation is mostly outside the eccentricity we imaged in V1.) The distance between the flanker and CE in the stimulus is 0.75 deg and the distance between the corresponding activation patches over V1 is about 6 mm, which is the expected distance by the magnification factor at this eccentricity [41]–[44]. To further study the VSDI response evoked by the CE and flanker in V1 and V2 we defined regions-of-interest (ROIs) in the evoked activation patches. For this purpose we selected pixels crossing a signal-to-noise ratio (SNR) threshold of the evoked activity (equation 1; see *Material and Methods*). In area V1 we could further fit 2D-Gaussians for the elliptical activation patches (Fig. 1B). We defined two ROIs in V1 (Fig. 1D-E; Only pixels within the top 10% of the fitted Gaussian area were defined as the ROI. see *Material and Methods*): V1-CE (area of central element in V1) and V1-flanker (area of flanker element in V1). We note that a vertically or horizontally oriented Gabor, evoked similar, highly overlapping activation patterns in V1. When fitting a 2D Gaussian, 95% of the pixels were overlapping between the ROIs defined for a horizontal or a vertical Gabor (Fig. 1A, B middle and bottom panels). Therefore, the ROIs in V1 were not affected by the orientation of the Gabor. This implies that a vertical or horizontal Gabor evoked similar activation patches in V1 and we did not observe any spatial bias due to the Gabor orientation (see *Discussion*). Moreover, we also studied a smaller ROI for the V1-CE. This smaller ROI is less affected by the adjacent flankers and represents a more focal part in the CE. Taking only pixels in the top 5% or 2.5% of the fitted 2D-Gaussian fit (instead of 10% used in the study) preserved the results. We also defined two corresponding ROIs in V2 (Fig. 1D-E): V2-CE (area of central element in V2) and V2-flanker (area of flanker element in V2). Thus the imaged area could be divided into well-defined regions in V1 and V2, each activated by a single Gabor, either the CE or the flanker [28].

### VSDI α-power is a Discriminative Measure at the Single Trial Level

**Figure 2:**
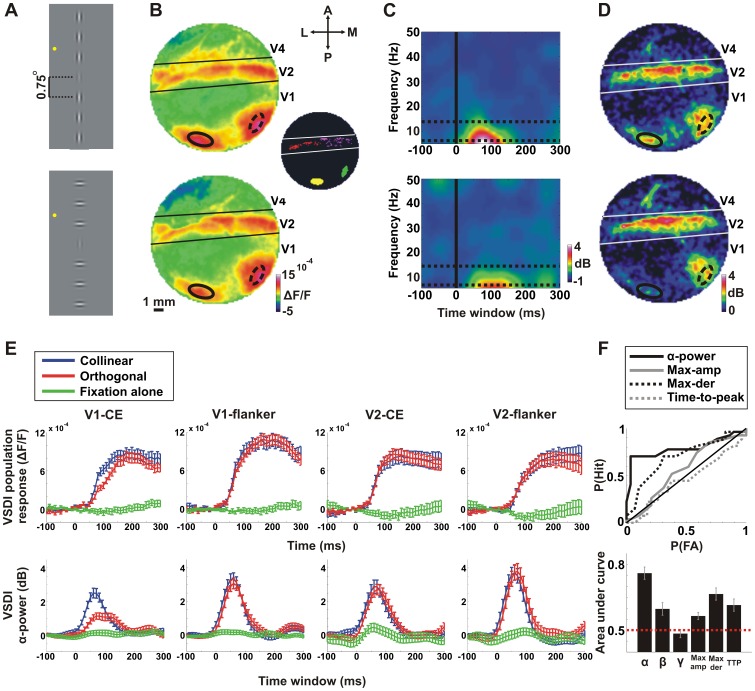
Spectrograms and α-power analysis of the VSDI signal. Data are from a representative recording session except for F, lower panel. *A*: Schematic illustration of the visual stimuli conditions: collinear (top) and orthogonal (bottom) conditions in which a central Gabor element (CE) was displayed at 16% contrast and the flanker Gabors were displayed at 64% contrast. The distance between Gabors was set at 0.75° (3λ). Fixation point is marked with a yellow dot. *B*: Population response (VSDI amplitude) maps evoked by the stimuli in A. Maps and stimuli in A are presented in a corresponding order (from top to bottom). The maps were averaged at 100–200 ms after stimulus onset. Color denotes normalized fluorescence change (ΔF/F). The ROIs of the V1-CE and V1-flanker are superimposed on the maps (solid and dashed black lines, respectively). The inset shows the 4 analyzed ROIs: V1-CE (yellow), V1-flanker (green) and V2-CE (red), V2-flanker (purple). *C*: VSDI spectrogram for the collinear (top) and orthogonal (bottom) conditions averaged over pixels in V1-CE. Color denotes power in dB. Time 0 is stimulus onset. Frequency range is 6–50 Hz (see *Materials and Methods*). The α-band is confined between two horizontal dashed lines. *D*: Average α-power maps (averaged 0–100 ms time window) for the collinear (top) and orthogonal (bottom) conditions. Color denotes α-power in dB. The spatial patterns of the α-power are corresponding to the population response maps depicted in B. *E*: *Top row*: VSDI population response for the collinear (blue), orthogonal (red) and fixation alone (green) conditions for the 4 ROIs (from left to right): V1-CE, V1-flanker, V2-CE and V2-flanker. Error bars indicate mean±SEM over trials (n = 27, 29 and 28 trials for the collinear, orthogonal and fixation alone conditions respectively). *Bottom row*: The same as the top row but for the VSDI α-power. *F:* A receiver-operating characteristic (ROC) analysis on collinear vs. orthogonal single trials for different neural population measures: average α-power (0–100 ms); average β-power (0–100 ms), average γ-power (0–100 ms), maximal population response, maximal derivative of the population response and time-to-peak of the population response. Top panel: the ROC curves of most population measures for a typical recording session. Bottom panel: the AUC for each neuronal population measure was calculated in each recording session separately and then averaged across all recording sessions. Error bars indicate mean±SEM over recording sessions (n = 8 recording sessions).

Following the retinotopic mapping, the monkeys were presented during fixation with a collinear or orthogonal stimulus (Fig. 2A; the CE and flankers were at 16% and 64% contrasts correspondingly; [10]). We confirmed that the response maps in Figure 2B showed the activation patches for the CE and the flanker in V1 and V2.

In a previous study we showed that the population response to contextual effects occurred within the first 200 ms after visual stimulus onset [28], thus we mostly analyzed the VSDI signal within this period. The first step in our analysis was to assess the time-frequency power content. To achieve this we calculated the spectrograms separately for the collinear and orthogonal stimuli (equations 2,3). Spectrograms were calculated using a 160 ms sliding Hamming window for each trial and pixel in the imaged area (minimal frequency is 6 Hz; see *Materials and Methods*). We then averaged the spectrograms across trials thus resulting in a time plot of power for each frequency and each pixel. Fig. 2C displays the spectrogram for the collinear (top) and orthogonal (bottom) conditions averaged over pixels in the ROI of the V1-CE. In accordance with previous publications [38], we found that the VSDI signal showed power mainly in the lower frequencies. Further analysis showed that the evoked frequency content of the VSDI signal shows mainly significant power in the α band (7–14 Hz; between the two dashed horizontal lines in Fig. 2C) compared to the fixation alone condition (p<0.001; Mann-Whitney U-test; n = 8 recording sessions; averaged 0–100 ms after stimulus onset) and much lower power in the beta band (15–24 Hz) that is only slightly higher yet significant compared to the fixation alone condition (p<0.05). Power in the gamma band (25–50 Hz) was not statistically different from the fixation alone condition. Thus, the VSDI signal contains mainly a large stimulus evoked α-power.

Fig. 2D depicts the α-power map, which is the time averaged (0–100 ms after stimulus onset) α-power for each pixel in the imaged area for the collinear (top) and orthogonal (bottom) conditions (equations 4,5). Comparing α-power maps with the corresponding population response maps in Fig. 2B clearly shows that the patches of high population response in V1 and V2 largely overlapped the patches of high α-power in both conditions.

When comparing between collinear and orthogonal conditions, the ROI of V1-CE showed faster dynamics and steeper rising phase of population response in the collinear than in the orthogonal condition [28]. This transient was corresponding temporally with higher α-power in the collinear condition (compare Fig. 2E two left panels). In contrast, there were no apparent differences between the stimulus conditions in the V1-flanker, V2-CE or V2-flanker ROIs for both population response and α-power measures (Fig. 2E).

To further study whether the α-power or other measures of the population response can discriminate between the two conditions at the single trial level, we performed a receiver-operating characteristic (ROC) analysis on collinear vs. orthogonal single trials. We compared the ROC curve for the average α-power (0–100 ms after stimulus onset) and additional neural population measures that were computed in the ROI of V1-CE. The additional neural population measures were: average beta power (β; 15–25 Hz; 0–100 ms), average gamma power (γ; 26–50 Hz; 0–100 ms), maximal population response, maximal derivative of the population response and time-to-peak of the population response. The ROC curves of most measures are shown in Fig. 2F (top panel; for a typical recording session). The area under the curve (AUC) quantifies the accuracy of the ROC classifier. AUC was calculated for each recording session separately and then averaged across all recording sessions. AUC was significantly higher for α-power (AUC = 0.76±0.03; Mean±SEM) when compared to other neural population measures (Fig. 2F bottom panel; AUC = 0.60±0.03, 0.48±0.02, 0.56±0.02 and 0.61±0.03 for average β-power, γ-power, maximal population response and time-to-peak of the population response correspondingly; Mann-Whitney U-test p<0.05 Bonferroni corrected). Finally, the AUC α-power was higher than the AUC for maximal derivative of the population response (0.67±0.03), however, this was not statistically significant (p = 0.09, Bonferroni corrected).

To investigate whether these effects are driven directly by the stimulus or whether part of the evoked response reflects internal cortical processing, we repeated this analysis after subtracting the mean stimulus-evoked response from each trial and each pixel. We therefore calculated the α-power, and the different neural population measures and the AUC values after removal of mean stimulus response. We found that AUC for the α-power was still higher than other measures although not statistically different. (AUC = 0.61±0.04 for α-power compared to AUC = 0.56±0.04, 0.49±0.03, 0.51±0.01, 0.54±0.02 and 0.49±0.02, for β-power, γ-power, maximal population response, maximal derivative and time-to-peak of the population response correspondingly; Mann-Whitney U-test; p = 0.06 with maximal population response and time-to-peak and p>0.06 for all other measures; Bonferroni corrected).

In summary, α-power was superior in discriminating between collinear and orthogonal conditions at the single trial level, compared to other frequency bands and population response measures (α-power was more discriminative than max-derivative but this was not statistically significant). These results indicate that α-power (in V1-CE ROI) is a relevant population measure and can be useful at the single trial level. Our next step was to examine whether VSDI α-coherence (i.e. synchrony rather than amplitude) between different areas in V1 and between V1 and V2 can reveal any differences between the conditions.

### VSDI α-coherence in V1 and V2 Areas, before and after Removal of Stimulus Contribution

We calculated the α-coherence of the population response (VSDI signal) in areas V1 and V2 (see *Material and Methods*). Coherence is a sensitive measure for estimating the linear correlation between two signals at each frequency independently, where 1 indicates perfect correlation and 0 indicates no correlation. Since α-power showed differences among conditions mainly in V1-CE, we first calculated the average α-coherence between population responses of all pixels in the V1-CE ROI and each pixel in the imaged area. This resulted in an α-coherence map where each pixel in the map represents the average α-coherence (as a function of time) between that pixel and all the pixels in the ROI of V1-CE (equations 6–10; and see *Material and Methods*). This was done for the collinear and orthogonal conditions separately.

**Figure 3:**
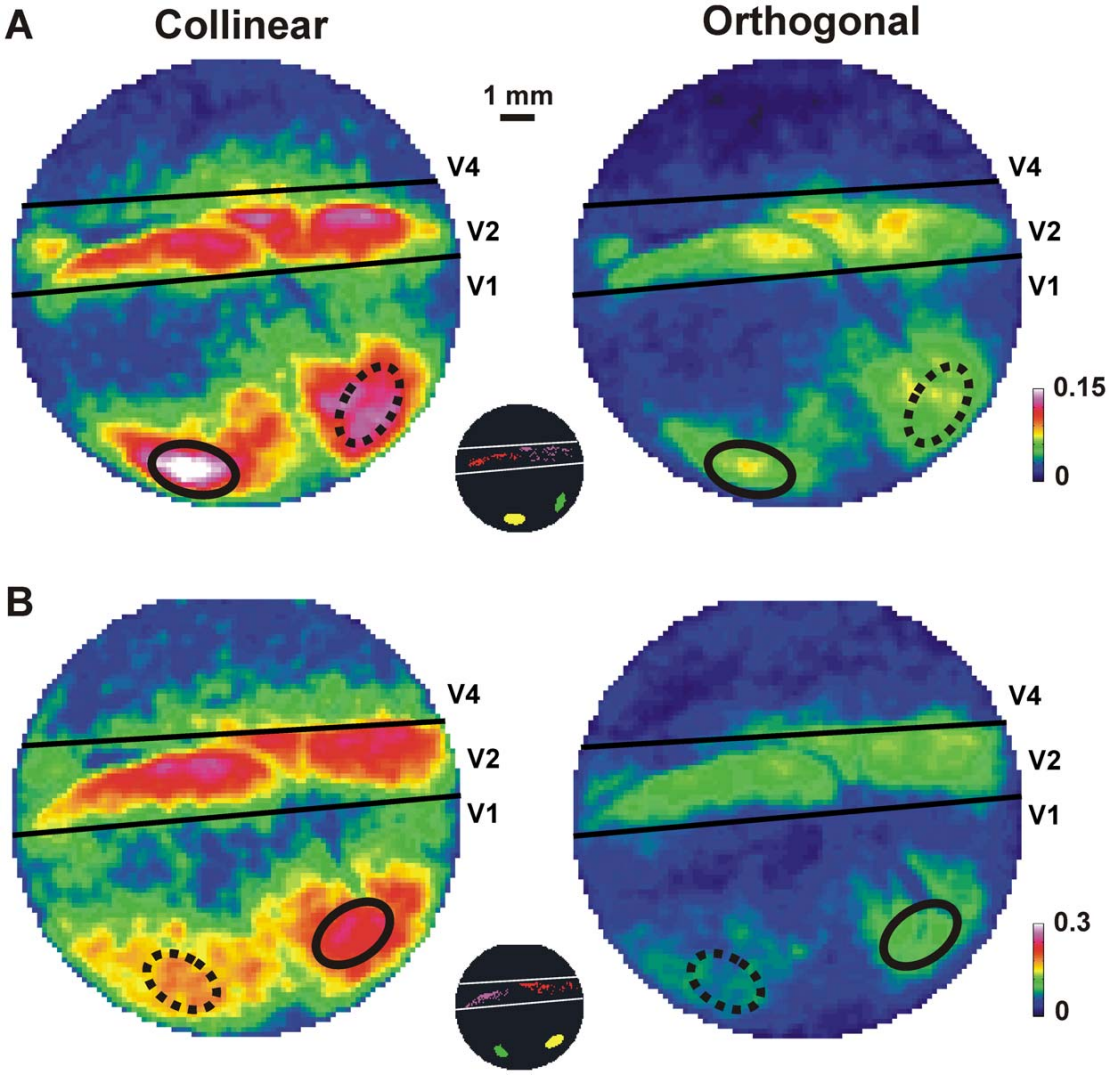
α-coherence maps in collinear and orthogonal conditions. *A*: Average α-coherence (AAC; averaged over 0–100 ms after stimulus onset) maps in the collinear (left) and orthogonal (right) conditions in one recording session. Each pixel in the map reflects the average AAC of that pixel with the ROI of V1-CE in the collinear (left) and orthogonal (right) conditions. Color denotes coherence values. The ROI of V1-CE (solid black ellipse) and the ROI of V1-flanker (dashed black ellipse) are superimposed on all maps. Inset indicates the 4 ROIs: V1-CE (yellow), V1-flanker (green), V2-CE (red) and V2-flanker (purple) for this recording session. *B*: AAC maps as in A, but for a different recording session with changed positions of CE and flanker.

Fig. 3 shows typical average α-coherence maps (AAC; averaged over time windows 0–100 ms after stimulus onset) for the collinear (Fig. 3A, left) and the orthogonal (Fig. 3A right) conditions in one recording session. The collinear condition showed clearly higher AAC values in both V1 and V2 areas than the orthogonal condition. The pixels with high AAC were not distributed homogeneously in V1 and V2, but were localized to V1-CE, V1-flanker, V2-CE and V2-flanker activated areas. Fig. 3B shows another recording session with changed locations of the CE and flanker stimuli. The increased AAC values for the collinear condition are evident.

**Figure 4:**
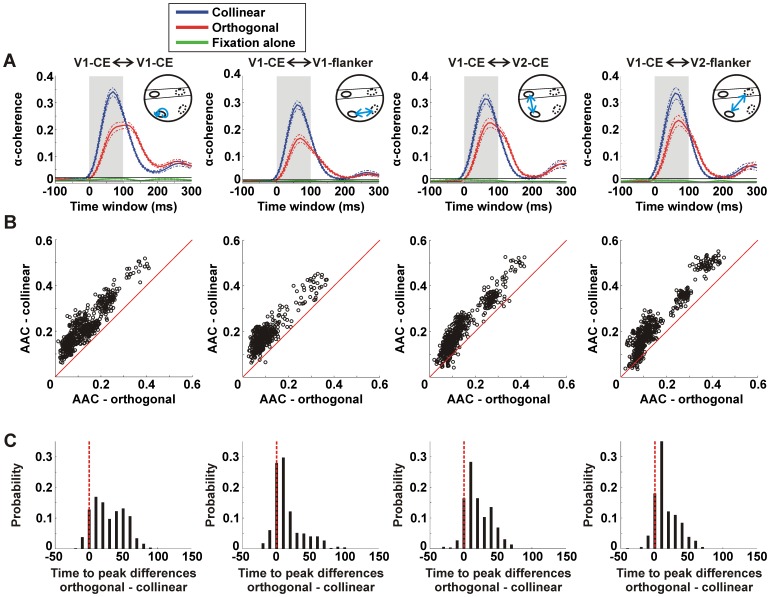
α-coherence dynamics in collinear and orthogonal conditions. *A*: α-coherence as a function of time between V1-CE and other ROIs for the collinear (blue), orthogonal (red) and fixation alone (green) conditions averaged over pixels within each of the 4 ROIs (from left to right): V1-CE (n = 555 pixels), V1-flanker (n = 444 pixels), V2-CE (n = 523 pixels) and V2-Flanker (n = 545 pixels). Dashed lines indicate mean±3×SEM. Black horizontal lines indicate upper and lower limits of the α-coherence (mean±3×SEM) in the fixation alone condition. A change in α-coherence is defined by exceeding these limits. There was significantly higher stimulus-induced α-coherence in both collinear and orthogonal conditions compared to the fixation alone condition (Mann-Whitney U-test; p<0.01; averaged 0–100 ms). The inset depicts the pair of ROIs (blue arrow) from which α-coherence was calculated (see Fig. 1E). *B*: Scatter plots of the average α-coherence (AAC) averaged 0–100 ms after stimulus onset (shaded bar in A) in the collinear (y-axis) vs. the orthogonal (x-axis) conditions for each pixel in the different ROIs as in A. Each point reflects the average α-coherence of all pixel pairs between this specific pixel (in a specific ROI) and the V1-CE ROI pixels (equation 8). Almost all pixels are above the diagonal meaning there is higher AAC in the collinear than the orthogonal condition. *C*: Histograms of time-to-peak (TTP) differences (orthogonal - collinear) of the α-coherence for the all pixels in the four ROIs as in B. Time = 0 indicates that peak α-coherence was reached at the same time window in both conditions. There is significantly lower TTP in the collinear than the orthogonal condition in each ROI (p<0.001). Data is from 8 recording session and 2 monkeys.

Next, we examined the α-coherence between the ROI of V1-CE and each of the four different ROIs: V1-CE, V1-flanker, V2-CE and V2-flanker. Fig. 4A shows the time course of the α-coherence between the ROI of V1-CE and each of the four ROIs for the collinear (blue), orthogonal (red) and fixation alone (green) conditions, averaged over all pixels and all recording sessions from both monkeys. We used the maximal and minimal values (mean±3×SEM; time window: -100 to 300 ms relative to stimulus onset) of the α-coherence in the fixation alone condition to set the upper and lower limits of α-coherence distribution (Fig. 4A; black horizontal lines). A change in stimulus-induced α-coherence was defined by exceeding these limits. The stimulus-induced α-coherence in both collinear and orthogonal conditions increased significantly in all ROIs compared to the fixation alone condition (Mann-Whitney U-test compared with fixation alone condition; p<0.001; averaged 0–100 ms). The collinear condition (blue curve) was also much higher than in the orthogonal condition (red curve) in all ROIs.

To quantify these results we plotted the AAC for every pixel in each recording session in a two dimensional space (collinear, y-axis and orthogonal, x-axis). This was done separately for each of the four ROIs. Fig. 4B shows higher AAC values in the collinear than in the orthogonal condition (almost all pixels appear above the diagonal). This result was significant in all ROIs, in each recording session separately and in all sessions pooled together (Mann-Whitney U-test, p<0.001).

The time-to-peak (TTP) of the α-coherence was shorter for the collinear than for the orthogonal condition. The histograms of TTP differences between the orthogonal and collinear conditions (Fig. 4C) show a clear bias for longer TTP values in the orthogonal condition (Mann-Whitney U-test; p<0.001 in all ROIs in each recording session separately, and in all sessions pooled together).

To investigate whether these effects are driven directly by the stimulus or whether part of the response reflects internal cortical processing, we repeated these analyses after subtracting the mean stimulus-evoked response from each trial and pixel, before calculating α-coherence (see *Materials and Methods*). Although clearly the AAC were now much lower (because we removed the direct stimulus contribution), we obtained highly similar and significant results in favor of the collinear condition (compare Figs. 3 and 4 with Figs. S2 and S3; see *Discussion*).

**Figure 5:**
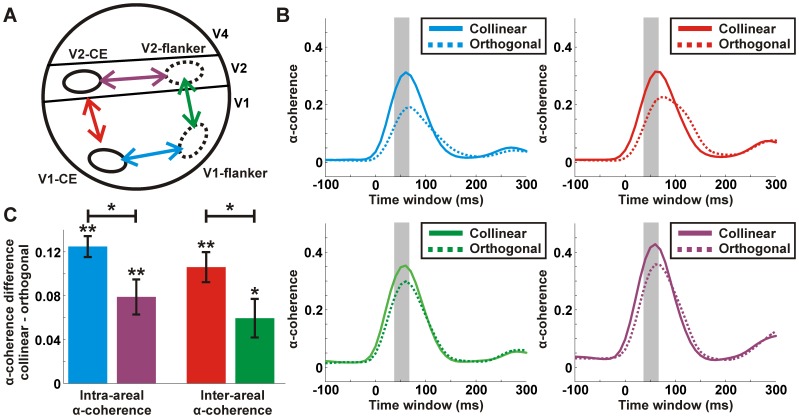
α-coherence between different ROIs in V1 and V2. *A:* A schematic illustration of the 4 ROIs used in this study: V1-CE, V1-flanker, V2-CE and V2-flanker. Superimposed are 4 different interactions each representing a pair of ROIs: V1-CE and V1-flanker (blue), V1-CE and V2-CE (red), V1-flanker and V2-flanker (green), V2-CE and V2-flanker (purple). *B:* The α-coherence as a function of time for the collinear (solid lines) and orthogonal (dashed lines) conditions calculated between each of the four pairs in A. *C:* α-coherence difference (collinear – orthogonal) between each of the four pairs in A. α-coherence was average 40–60 ms after stimulus onset (shaded bar in B). Error bars indicate SEM over recording sessions (n = 8 recording session from 2 monkeys). Asterisks denote significant α-coherence compared to 0 or between pairs. * p<0.05; ** p<0.01.

Fig. 5 depicts the α-coherence between different pairs of ROIs. α-coherence between two given ROIs was calculated by averaging the α-coherence for all the possible pixel-pairs between the ROIs (see *Materials and Methods*). We present the α-coherence between 4 different ROIs-pairs: V1-CE with V2-CE; V1-CE with V1-flanker; V1-flanker with V2-flanker; V2-CE with V2-flanker (Fig. 5A). Fig. 5B displays the α-coherence as a function of time for each of the 4 pairs. In all pairs α-coherence is higher in the collinear condition compared to the orthogonal condition. The difference in α-coherence between conditions was significantly higher than 0 for each pair separately (Fig. 5C; sign ranked test; p<0.05). Similar results were obtained after subtracting the mean stimulus-evoked response. In addition we find that intra-areal V1-V1, namely CE-flanker, displayed significantly higher α-coherence compared to intra-areal V2-V2, and inter-areal V1-V2 in the CE displays significantly higher α-coherence compared to inter-areal V1-V2 flanker (Mann-Whitney U-test; p<0.05).

We note that other population measures, e.g. population response and α–power, showed no differences between collinear and orthogonal conditions in the V1-flanker, V2-CE and V2-flanker (Fig. 2E). Hence, we infer that an increase in synchronization rather than response amplitude is related to collinear effects (see *Discussion*). Finally we note that the above spatio-temporal patterns cannot result from microsaccades, as the results were verified in trials lacking microsaccades.

In summary, our analysis showed that the collinear condition was characterized by increased intra-areal and inter-areal α-coherence with shorter latencies. The higher α-coherence analysis revealed a population network which is characterized by synchrony, rather than basic population measures, hence suggesting that synchrony plays an important role in mediating contextual effects.

## Discussion

We have studied the relation of synchronization to collinear effects in V1 and V2 areas using voltage-sensitive dye imaging (VSDI). Time-frequency analysis enabled studying different spectral components of the VSDI signal (above 6 Hz), revealing significant stimulus-evoked α-power, much lower β-power and no significant stimulus-evoked γ-power. We found higher and faster intra-areal and inter-areal α-coherence between the neural populations processing individual Gabor patches in the collinear condition than in the orthogonal condition.

### VSDI α-band as an Informative Population Measure

The VSDI signal contains a significant stimulus-evoked α-power. How is this measure different from or better than other, more basic population measures? We showed that α- power is more discriminative between collinear and orthogonal stimuli than other population measures at the single trial level (Fig. 2). When removing the stimulus contribution, the AUC values of the α-power were still larger than for other neural population measures suggesting that α-power may reflect also internal cortical processing (see also section below). We suggest that only a part of the frequencies comprising the VSDI signal, i.e. the α-power band, carry information on collinear interactions, whereas other frequency components, i.e. beta and gamma bands are much less modulated by collinear interactions.

### α-coherence before and after Removal of the Stimulus-locked Activity

To investigate the internal cortical synchronization between neuronal populations, namely co-varied activity that is not directly induced by the stimulus [45], previous studies removed the stimulus-locked contribution from a signal before calculating its coherence [25], [46]. We did this by subtracting the mean stimulus-evoked response from each trial and pixel before calculating coherence. α-coherence before and after removing the mean stimulus-evoked response gave similar results, but with a much larger difference between conditions before removing the mean response. This similarity can be interpreted in two ways: (i) the subtraction procedure does not completely eliminate stimulus-locked activity or (ii) stimulus-locked activity and internal processing of the stimulus within the cortical network are interwoven. We note that removing the stimulus locked contribution is not justified in all cases and two reasons that should be considered for our stimulus conditions are: i) the central element was identical in both the collinear and orthogonal conditions, implying that the stimulus contribution to α-coherence should be equal in both conditions and should not lead to higher α-coherence in the collinear condition. ii) Internal cortical processing, as manifested by correlated activity after removing the stimulus contribution, could still be time-locked to the stimulus, particularly when presenting a short stimulus (200 ms). If so, then removing the mean response may also eliminate to some extent, internal cortical processing. In conclusion, we propose that the internal cortical processing of the stimulus is partially time-locked to stimulus presentation making it difficult to separate between internal cortical correlations and direct stimulus-locked correlations.

### The Role of Intra-areal and Inter-areal Neural Synchronization in Collinearity and Relation to Behavior

Previous studies have shown that firing rate in V1 is enhanced under collinearity [8], [22], [24], [25], [47]–[49], however the role of synchrony remained unclear [25], [28]. In particular, the role of synchrony between V1 and V2 areas in collinear condition has not been studied. We found higher synchronization with collinearity (manifested as α-coherence) starting earlier among retinotopically matching regions in V1 and V2. A synchronized intra-areal and inter-areal network of activation is evident in the collinear condition relatively early after stimulus onset (Fig. 5). This may also correspond to the “pre-attentive” characteristics of collinear stimuli. Importantly, although in our previous study we found population response differences in the rising phase of the VSDI signal in the V1-CE area [28], there were no population response differences in V1-flanker areas and V2 areas. These findings further support the binding-by-synchrony theory at the population level: good continuation results in synchronized activity among neuronal populations without modulation or enhancement of activation rate [29], [30]. Finally, we note that Pooresmaeili et al. [14] showed that the flanker reduced the firing rate of neurons processing the CE rather than increasing it. We emphasize that our study concentrates mainly on collinear versus orthogonal effects rather than comparing the effects of CE with or without flankers. We observe enhanced population activity in the V1-CE ROI for the collinear condition compared to the orthogonal condition which can be attributed to a contextual influence mediated by horizontal connections. Although we cannot rule out completely the possibility that these influences may result from a spatial bias of a vertical flanker compared to the horizontal flanker, there is some evidence against this notion. Similar to our analysis, previous studies have shown that the activation patch of a single Gabor did not show anisotropy in favor of the Gabor’s collinear axis whereas the distribution of horizontal connection did [47]. We set the distance between the CE and the flankers (3λ) in accordance with previous psychophysical and electrophysiological studies [10]–[14], [28]. It has been shown that at this distance single unit activity is barely enhanced at the V1-CE when presenting only the flankers [14] whereas the VSDI signal, reflecting mainly subthreshold activity, does show increased response amplitude [28]. Sub-threshold activity outside the classical receptive field has been shown to be mediated by horizontal connections [50]. Similar subthreshold activity was induced at much larger separation distances between flankers and CE up to 7λ [28]. Thus, we suggest that the increase in VSDI response amplitude in the V1-CE when presenting flankers is more likely to result from collinear influences propagating by horizontal connections.

Although we find a highly synchronized network between V1 and V2 areas processing the CE and flanker, the level of synchrony was different among the four ROIs. We found two ROI interactions displaying higher synchrony compared to others: (1) intra-areal synchronization in V1 between CE and flanker elements (2) inter-areal synchronization between V1-CE and V2-CE. The former interaction may reflect the collinear effect in V1 whereas the latter may reflect the feed-forward propagation of this information from V1 to V2. Both interactions may lead to an enhancement in target visibility resulting in target facilitation.

In our study the monkeys were trained on a passive fixation task and were not required for a perceptual report of the central element. Thus, the direct behavioral effects of the central element detection in collinear and orthogonal array were not assessed in our study. Nevertheless, the difference in neural synchronization that we observed in V1, suggests that this measure can link between cortical activity and Gestalt laws and may imply a further link with behavior. A similar approach was demonstrated recently by Jancke et al. [51] who used VSDI to demonstrate the putative contribution of cortical activity to perceptual correlates of the line motion illusion in anaesthetized cats. Our monkeys were presented with a central Gabor at a contrast above threshold detection in humans (16% contrast compared to ∼11% contrast in [10]). This threshold was chosen to obtain a reasonable VSDI response to the target alone condition. Future experiments are required to determine whether our findings also hold for stimuli near perception threshold.

Our results differ from those of Roelfsema et al. [25] who recorded multi-unit activity (MUA) in behaving monkeys during a contour-grouping task. They concluded that synchrony was unrelated to contour grouping. There may be several reasons for this discrepancy. First, apart from behavioral paradigm and stimuli differences, they used a relatively wide window of 400 ms for noise correlation (mostly 200–600 ms, but also 0–400 ms after stimulus onset). This can either exclude the initial transient response or “dilute” it within the wide time window (e.g. 0–400 ms; Fig. 1B in [25]). Our results explicitly showed synchrony (in terms of coherence) differences in the initial transient response and, thus, synchrony effects may be present exclusively in the rising phase of the evoked response. Second, Roeflsema et al. [25] measured synchrony by calculating the cross-correlation in time between multi-unit activities on the same contour. As we showed, possibly only a small and specific component of the signal (i.e., α-band) was synchronized among sites, whereas other components were not. Thus, the latter may mask the synchronized components and interfere with synchronization analysis.

Using spectral analysis, we decomposed the VSDI signal into different frequency bands and found that only the α-band showed increased synchrony (α-coherence) in the collinear condition. We also compared the α-coherence to temporal correlation of the raw signal (i.e. without any filtration) and found that it showed stronger and more significant differences between collinear and orthogonal conditions. It also was more consistent over recording sessions compared to temporal correlations (data not shown). This was especially true in V2 areas where the VSDI signal was more prominent to heart beat noise due to the proximity of the large blood vessels in the lunate sulcus. This suggests that focusing on the alpha band coherence enabled studying synchronization, between and within areas, in a more reliable manner in the VSDI signal.

Intrinsic V1 synchrony can be mediated by horizontal connections in perceptual grouping, as suggested previously [8], [20], [22], [24], [25], [47], [52]. V1-V2 synchrony may be mediated by feed-forward or feed-back connections [26], [53], [54]. Although V1-V2 synchrony has been found relatively early after stimulus onset, implying a feed-forward role, Hupe et al. [55] and Li et al. [56] showed that feedback modulation can occur very early after stimulus onset. In this study, we used a 160 ms time window, resulting in a sub-optimal temporal resolution to decipher this problem.

We conclude that collinearity can be mediated by a synchronized network distributed within and across early visual areas, hence binding together discrete elements which may lead to perceptual binding and facilitation in target detection. Additional experiments and analysis are required to study whether synchronization plays an important role in more complex and noisy visual scenes where a contour needs to be segregated from a noisy background.

## Materials and Methods

### Behavioral Task and Visual Stimuli

We obtained data from 8 VSDI recording sessions from two hemispheres of two monkeys (*Macaca fascicularis,* males, L and A, 7–11 Kg). The monkeys’ water, food consumption and weight were checked daily and their health was monitored by a veterinarian. All procedures were in accordance with *National Institutes of Health Guide for the Care and Use of Laboratory Animals, Bar-Ilan University Guidelines for the Use and Care of Laboratory Animals in Research* and the recommendations of the *Weatherall report*. All procedures were approved and supervised by the *Institutional Animal Care and Use Committee (IACUC)*. These procedures were approved by the National Committee for Experiments on Laboratory Animals at the Ministry of Health (permit number 36-12-04).

The monkeys were trained on a fixation task while collinear or orthogonal stimuli of Gabor arrays were presented. The monkeys were required to maintain fixation within a small fixation window (±1 deg) for a random interval (3–4 sec). Next, a visual stimulus was displayed for 200 ms and the monkeys were required to maintain fixation for another 2 sec. They were rewarded only if they fulfilled these requirements (correct trials). In this task we used three conditions, in two of which Gabor arrays were presented: 1) the *collinear condition* (Fig. 2A top): a low-contrast (16%) central element (CE) with 1 or 3 high-contrast (64%) flankers from each side, orientated similarly to the CE, 2) the *orthogonal condition* (Fig. 2A bottom): a low-contrast CE with 1 or 3 high-contrast flankers from each side, orientated at 90° to the CE. 3) The *fixation alone condition*: no stimulus presentation. This condition was used to remove the heartbeat artifact (see *Basic analysis of VSDI signals*). Similar results were obtained for either 1 or 3 flankers (5 recording sessions with 3 flankers and 3 recording sessions with 1 flanker on each side of the CE). The distance between Gabors was set at 0.75° (3λ; λ is the wavelength of the Gabor) as in human psychophysical studies [10], [57]. Over the recording sessions the eccentricity of the CE Gabor varied from 0.9–2° below the horizontal meridian and 0.5–1° from the vertical meridian [28].

### Eye Position Monitoring

Eye position was monitored using an infrared eye tracker (Dr. Bouis Device, Kalsruhe, Germany), sampled at 1 kHz and recorded at 250 Hz. During the behavioral task the monkey was requested to maintain fixation on a small fixation point while visual stimuli were presented. Fixation was kept within ±1 deg throughout stimulus presentation.

We note that as we analyzed the VSDI signal mainly in the first 200 msec after stimulus onset, the number of eye movements made by the animals were very small. This is due to the low rate of microsaccades during fixation (the animals were well trained on fixation) and also due to the well established microsaccadic/saccadic inhibition effect following visual stimulus onset [58]. Nevertheless, the eye movement rate as a function of time did not differ between the collinear and orthogonal conditions.

### Surgical Procedures and VSD Imaging

The surgical procedure and voltage-sensitive dye staining have been reported in detail elsewhere [40], [59], [60]. Briefly, all surgeries were performed under general anesthesia using sodium pentobarbital or isoflurane. A head holder and two cranial windows (25 mm ID) were bilaterally placed over the primary visual cortices and cemented to the cranium with dental acrylic cement. A craniotomy was performed, and the dura mater was removed, exposing the visual cortex. A thin and transparent artificial dura made of silicone was implanted over the visual cortex. Appropriate analgesics and antibiotics were given during surgery and postoperatively as required. All surgeries and follow-ups were under the supervision of a veterinarian and all efforts were made to minimize suffering.

The center of the exposed and imaged V1 area lay 1–3° below the horizontal meridian and 1–2° from the vertical meridian. We stained the cortex with the voltage-sensitive dyes RH-1691 or RH-1838 (Optical Imaging, Israel). VSDI was performed using a Micam Ultima system with a sampling rate of 10 ms/frame (100 Hz) and a spatial resolution of 10,000 pixels/frame. Each pixel sums the neuronal activity from an area of approximately 170 µm×170 µm that includes a few hundred neurons. The protocol of data acquisition in VSDI has been described in detail elsewhere [28], [40].

### VSDI Basic Analysis

We analyzed only those trials with tight fixation, in which the monkey did not exceed the fixation window limits during stimulus presentation. VSDI trials in which the response exceeded the mean VSDI (across trials) response by two standard deviations on each side were suspected to include noisy trends and therefore were excluded from further analysis.

Each pixel in each trial was normalized to DC level (divided by baseline fluorescence just before stimulus onset) and a linear regression (fitted to baseline activity) was subtracted (detrending). This eliminated the slow fluctuations in the VSDI signal, e.g. the photo bleaching effect. To remove the heartbeat artifact, for each pixel, the mean response of the fixation alone condition was subtracted from each trial, pixel wise. These steps are schematically illustrated and explained in Ayzenshtat et al. [61] Supplementary Fig. 12.

### Definition of Regions of Interest

For each recording session we defined regions of interest (ROIs) in V1 and V2 areas based on the presentation of only the CE (Fig. 1B top) or just the flankers most proximal to the CE (either vertically or horizontally oriented; Fig. 1B middle and bottom). For this purpose we selected pixels crossing a signal-to-noise ratio (SNR) threshold of the evoked activity (equation 1; In our case, the absolute value was not necessary).
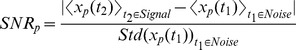
(1)where 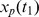 and 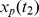 are mean time series (averaged over trials) of a given pixel *p*. *Signal* refers to the population response (t_2_; 50–100 ms *after* stimulus onset), and *noise* refers to the baseline activity (t_1_; 50–100 ms *before* stimulus onset). SNR units are expressed as standard deviation units. In area V1 we could further fit 2D-Gaussians for the elliptical activation patches (Fig. 1B). Pixels within the top 10% of the fitted Gaussian area were defined as the ROI [28]. We defined two ROIs in V1 (Fig. 1D-E): V1-CE (area of central element in V1) and V1-flanker (area of flanker element in V1). To study whether a vertical or horizontal Gabor may generate a spatial bias in our ROI definition we did the following: For the V1-flanker we fitted a 2D-Gaussian separately for either vertically or horizontally oriented flankers. In general activation maps for both orientations were very similar (Fig. 1B middle and bottom). We found that the V1-flanker ROI, defined by a horizontally or vertically oriented Gabor, was highly similar among the two orientations, i.e. above 95% of the ROIs pixels were overlapping in both cases. This implies that a vertical or horizontal Gabor evoked a similar activation patch in V1 and we did not observe any spatial bias due to the Gabor orientation [47]. VSDI activation over the exposed V2 area did not exhibit a Gaussian-like pattern (see Figs. 1B and 2B for examples; large parts of V2 area are buried within the Lunate Sulcus). V2 pixels activated by the CE Gabor with SNR exceeding a threshold of 5 STD were included in the ROI of V2-CE, whereas V2 pixels activated by the flanker Gabor (Fig. 1B middle and bottom) with SNR exceeding a threshold of 5 STD were included in the ROI of V2-flanker. Thus the imaged area could be divided into well-defined regions in V1 and V2, each activated by a single Gabor, either the CE or the flanker [28]. Fig. 1D-E depict the 4 different ROIs that were used in this study: V1-CE, V1-flanker, V2-CE and V2-flanker. The border between V1 and V2 was obtained by imaging the ocular dominance map [62] using optical imaging of intrinsic signals [63].

### Power Spectrum Analysis

To study the spectral content of the VSDI signal we calculated a short-time Fourier transform (STFT) using a 160 ms sliding Hamming window (VSDI signal was sampled at 100 Hz; step size 10 ms). This was done for each pixel in every trial separately (equations 2–3). Because the stimulus evoked VSDI signal is a non-stationary signal with transient responses, we used STFT rather than band-pass filtering. Fast Fourier Transform (FFT) was computed on each window (equation 2) after subtracting the mean population response of the window and multiplication by a Hamming window. Next, power was calculated for each frequency and averaged over trials, resulting in a frequency-time plot (spectrogram) for pixel *p* in the imaged area (equation 3).
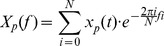
(2)

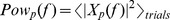
(3)Where: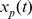 is the time series (*t = 1,…,N*; *N* = 16 time points) of a given window for pixel *p* in the imaging area. This is done for each and every trial. 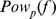 is the power function in a given window for pixel *p*. Note that the power function is the mean power across trials.

The outcome of this analysis was a power spatial map as a function of time for each frequency (equation 4). To obtain an α-power map (Fig. 2D), we averaged the power at frequencies in the α-band for each pixel (7–14 Hz; equation 5). To obtain the α-power in a given ROI we averaged the α-power over pixels belonging to the ROI (Fig. 2E bottom panels).
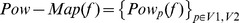
(4)

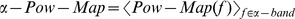
(5)


The raw power of the signal was normalized to the background activity (divided by the power before stimulus onset) and is shown in log scale (dB units). The 160 ms window was found to be the best tradeoff between better time resolution and more accurate evaluation of power frequency. This window limited our power analysis to frequencies above 6.25 Hz in both signals, whereas the upper limit is set by the Nyquist frequency (50 Hz). We obtained similar results using (1) different time windows (200 ms and 320 ms; data not shown) (2) a multi-taper method (open source Chronux http://chronux.org/; [64]) using parameters previously shown to generate similar results in the FFT analysis used here [65]. In addition, we calculated the spectrograms after subtracting the mean stimulus-evoked VSDI response from every trial and every pixel separately for each condition. We obtained similar spectrograms for both conditions (see Fig. S1) implying that even after the removal of the stimulus contribution and therefore, removing most of the transient response, the frequency content remains similar (as expected, after removal of stimulus contribution the power values were reduced).

### Coherence Analysis

To study correlation in the frequency domain, coherence was calculated in a 160 ms sliding window analysis. Coherence is a sensitive measure for estimating the linear correlation between two signals at each frequency independently. Obtaining a high coherence value at a given frequency requires a high co-variation of the spectral amplitude and a constant phase difference (not necessarily zero) at this frequency across trials [31]. We calculated coherence between different areas in V1 and V2 and compared between the collinear and orthogonal conditions.

We first focused on the coherence between the V1-CE ROI and other pixels in the V1 and V2 areas. For each frequency, we calculated a coherence map that reflects the coherence between pixels in V1-CE ROI and other pixels in V1 and V2. To do this, we first calculated the cross-spectra between any two pixels, one pixel in the V1-CE ROI and any other pixel belonging to the imaging area (equation 6) and then their coherence (equation 7). For each pixel in the imaging area we averaged the coherence values of all pairs between that pixel and all the pixels in the V1-CE ROI (equation 8). The group of average coherence values in all pixels in the imaged area depicts a coherence map that reflects the coherence between pixels in V1-CE ROI and other pixels in V1 and V2 (equation 9). Next we averaged coherence values over the α band to obtain an α-coherence map that reflects the α-coherence between pixels in V1-CE ROI and other pixels in V1 and V2 (equation 10; Fig. 3). Each pixel in the map reflects the average α-coherence of all pixel pairs between this specific pixel and the V1-CE ROI pixels (equation 8). For example, if there were 100 pixels in the V1-CE ROI, the α-coherence of one pixel in the map reflects the average α-coherence of 100 pairs between that pixel and all pixels in the V1-CE ROI. This was done for the collinear and orthogonal conditions separately.
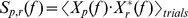
(6)

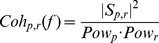
(7)

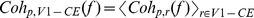
(8)

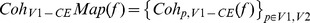
(9)

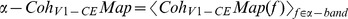
(10)Where: 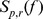 is the cross-spectra between pixels *p* and *r* in a given window. 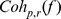 is the coherence function between pixels *p* and *r* in a given window. * denotes the complex conjugate. We note that coherence, unlike power and cross-spectra, is not a trial-wise measure since it is derived from the average (across trials) of power and cross-spectra of two pixels.

To calculate the average α-coherence between the V1-CE ROI and another ROI we averaged the V1-CE α-coherence values in pixels belonging to that ROI (Fig. 4A). By following the above analysis, we can obtain the average α-coherence between any pair of ROIs in the V1 and V2 areas (Fig. 5B). For example, if there were 100 pixels in the V1-CE ROI and 200 pixels in the V1-flanker ROI, then the α-coherence between both ROIs is the average α-coherence of all the 20,000 different pairs between the two ROIs.

By calculating coherence values on the signal itself we also include stimulus-locked activity that may contribute to high coherence levels without any coherent activity [45]. To remove the stimulus-locked contribution and avoid fast onset transients, we subtracted the mean stimulus-evoked VSDI response from every trial and every pixel separately for each condition. For example, the mean evoked response to the collinear condition was subtracted from each collinear trial. We obtained similar results for both calculations: before and after the removal of stimulus contribution (Compare Figs. 3 and 4 with S2 and S3) and we mainly focus on coherence analysis without the removal of stimulus contribution (see *Discussion*).

## Supporting Information

Figure S1 **VSDI spectrograms before and after subtracting the mean stimulus-evoked response from each trial and pixel.** The VSDI spectrogram in the collinear condition averaged over pixels in the V1-CE ROI before (A; as in Figure 2C top) and after (B) subtracting the mean stimulus-evoked response from each trial and pixel (see Materials and Methods). Color denotes power in dB. The two black dashed lines confine the α-band. Stimulus onset is at t = 0.
(TIF)

Figure S2 **α-coherence maps after removing the stimulus-locked contribution.** Similar to Figure 3 but here α-coherence was calculated after subtracting the mean stimulus-evoked response from each trial and pixel (see *Materials and Methods*). *A*: Average α-coherence (AAC; averaged over 0–100 ms after stimulus onset) maps in the collinear (left) and orthogonal (right) conditions in one recording session. Each pixel in the map reflects the average AAC of that pixel with the ROI of V1-CE in the collinear (left) and orthogonal (right) conditions. Color denotes coherence value. The ROI of V1-CE (solid black ellipse) and the ROI of V1-flanker (dashed black ellipse) are superimposed on all maps. Inset indicates the 4 ROIs: V1-CE (yellow), V1-flanker (green), V2-CE (red) and V2-flanker (purple). *B*: AAC maps as in A, but for a different recording session with different positions of CE and flanker.
(TIF)

Figure S3 **α-coherence dynamics after removing the stimulus-locked contribution.** Similar to Figure 4 but here α-coherence was calculated after subtracting the mean stimulus-evoked response from each trial and pixel (see *Materials and Methods*). *A*: α-coherence as a function of time for the collinear (blue), orthogonal (red) and fixation alone (green) conditions averaged over pixels within each of the 4 ROIs (from left to right): V1-CE (n = 555 pixels), V1-flanker (n = 444 pixels), V2-CE (n = 523 pixels) and V2-Flanker (n = 545 pixels). Dashed lines indicate mean±3×SEM. Black horizontal lines indicate upper and lower limits of the α-coherence (mean±3×SEM) in the fixation alone condition. A change in α-coherence is defined by exceeding these limits. There was a significantly higher stimulus-induced α-coherence in the collinear condition compared to the fixation alone condition (Mann-Whitney U-test; p<0.01; averaged 0–100 ms). The inset depicts the pair of ROIs (blue arrow) from which α-coherence was calculated (see Fig. 1E). *B*: Scatter plots of the average α-coherence (AAC) averaged 0–100 ms after stimulus onset, in the collinear (y-axis) vs. the orthogonal (x-axis) conditions for each pixel in the different ROIs as in A. Most pixels are above the diagonal meaning there is higher AAC in the collinear than the orthogonal condition (Mann-Whitney U-test; p<0.001 for each ROI separately). *C*: Histograms of time-to-peak (TTP) differences (orthogonal - collinear) of the α-coherence for the all pixel in the four ROIs as in B. Time = 0 indicates that peak α-coherence was reached at the same time window in both conditions. There is significantly lower TTP in the collinear than the orthogonal condition in each ROI (Sign ranked test; p<0.001 for each ROI separately). Data is from 8 recording session and 2 monkeys.
(TIF)
