# Supplementary figures and images for: Collinear Stimuli Induce Local and Cross-Areal Coherence in the Visual Cortex of Behaving Monkeys
Source: PLoS One. 2012 Nov 19;7(11):e49391. doi: 10.1371/journal.pone.0049391 (PMC3501522; doi:10.1371/journal.pone.0049391)

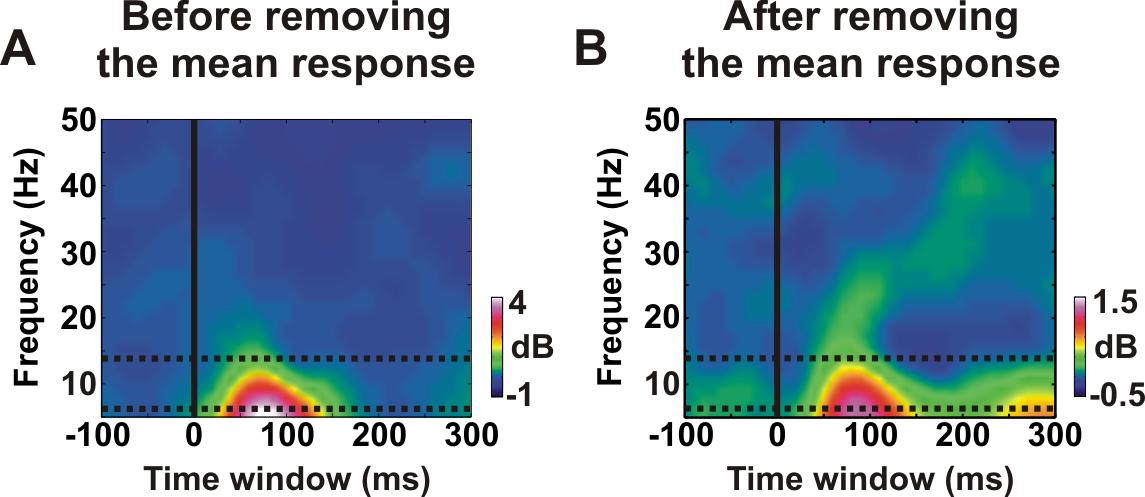

Supplement: Figure S1 — VSDI spectrograms before and after subtracting the mean stimulus-evoked response from each trial and pixel. The VSDI spectrogram in the collinear condition averaged over pixels in the V1-CE ROI before (A; as in Figure 2C top) and after (B) subtracting the mean stimulus-evoked response from each trial and pixel (see Materials and Methods). Color denotes power in dB. The two black dashed lines confine the α-band. Stimulus onset is at t = 0. (TIF) [file pone.0049391.s001.tif]

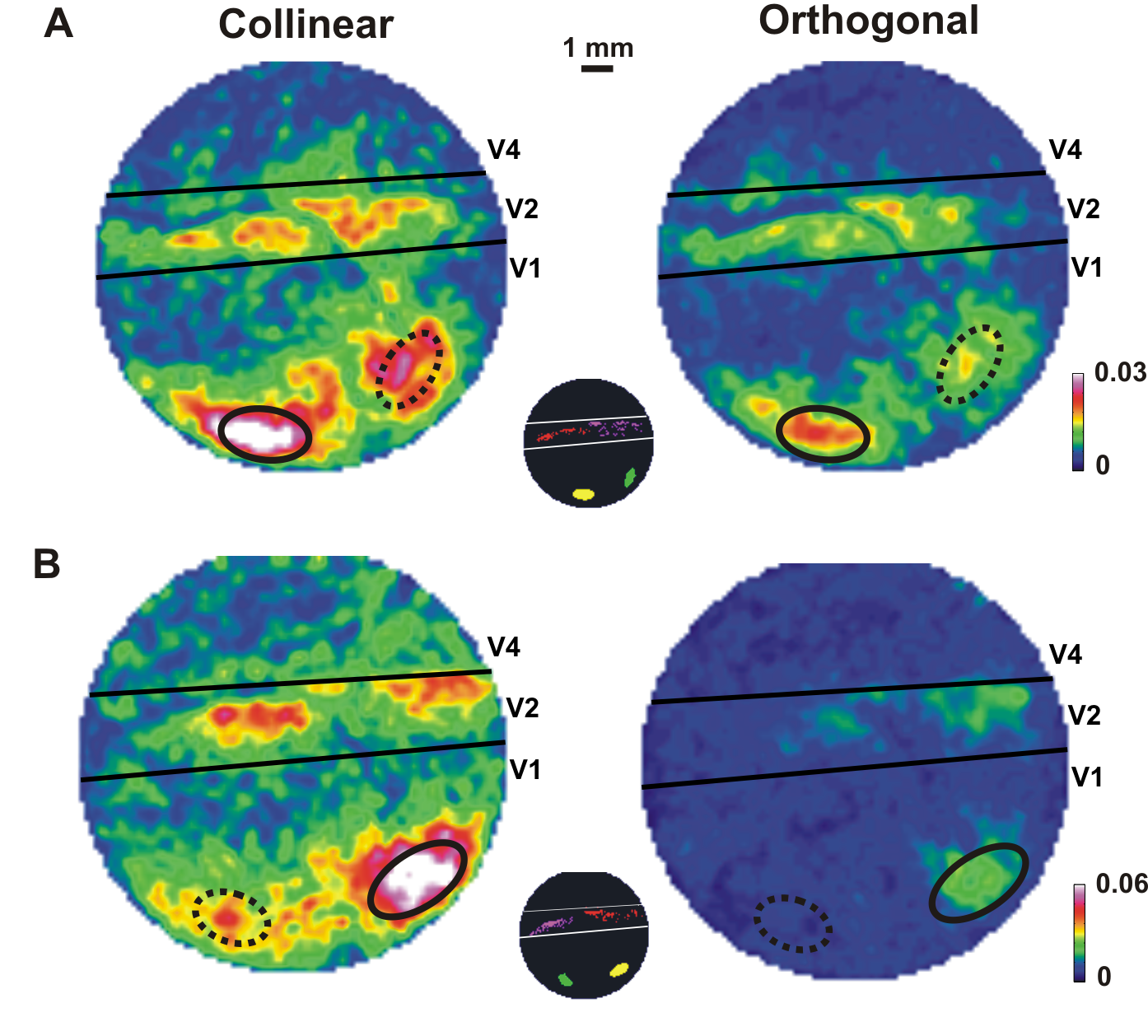

Supplement: Figure S2 — α-coherence maps after removing the stimulus-locked contribution. Similar to Figure 3 but here α-coherence was calculated after subtracting the mean stimulus-evoked response from each trial and pixel (see Materials and Methods). A: Average α-coherence (AAC; averaged over 0–100 ms after stimulus onset) maps in the collinear (left) and orthogonal (right) conditions in one recording session. Each pixel in the map reflects the average AAC of that pixel with the ROI of V1-CE in the collinear (left) and orthogonal (right) conditions. Color denotes coherence value. The ROI of V1-CE (solid black ellipse) and the ROI of V1-flanker (dashed black ellipse) are superimposed on all maps. Inset indicates the 4 ROIs: V1-CE (yellow), V1-flanker (green), V2-CE (red) and V2-flanker (purple). B: AAC maps as in A, but for a different recording session with different positions of CE and flanker. (TIF) [file pone.0049391.s002.tif]

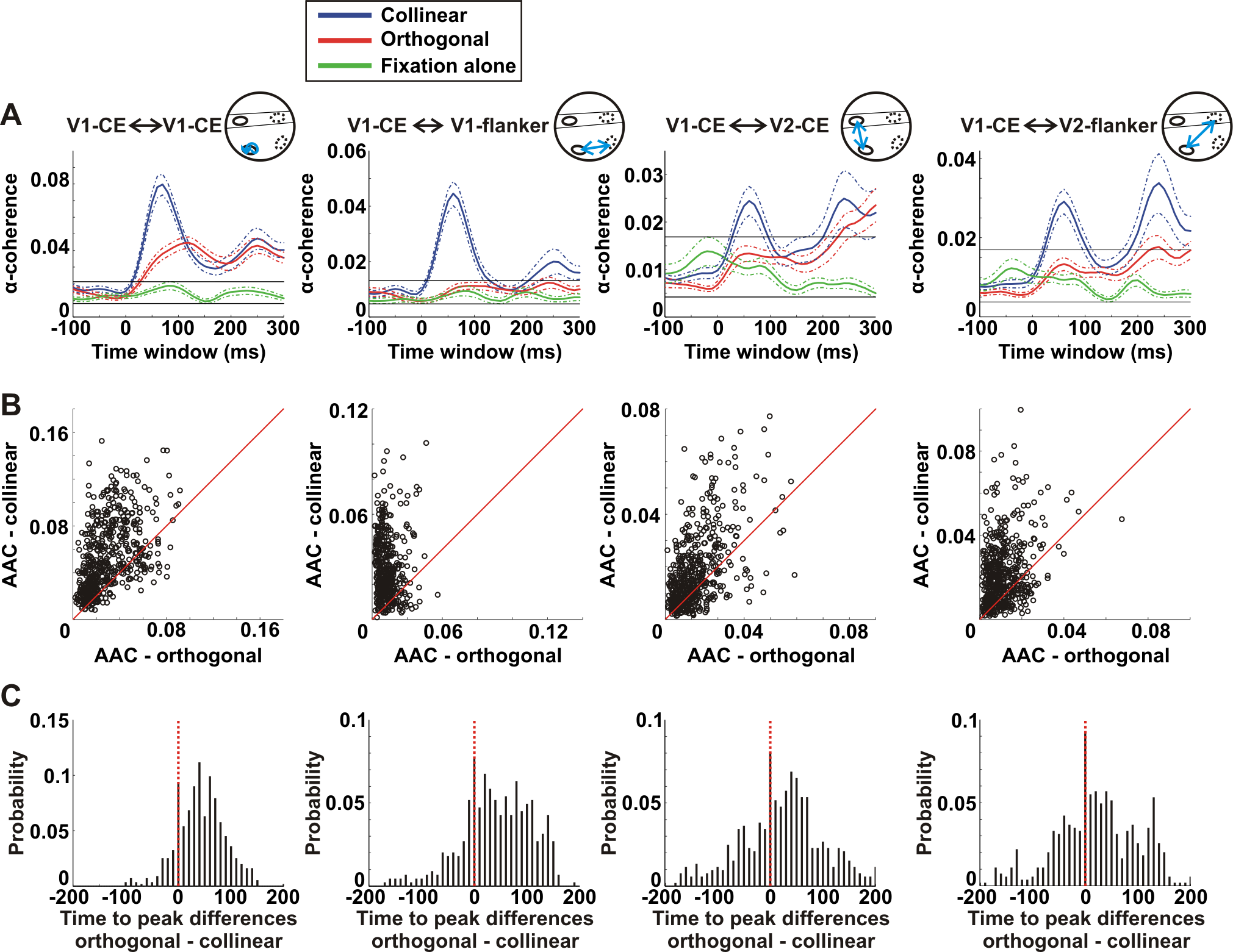

Supplement: Figure S3 — α-coherence dynamics after removing the stimulus-locked contribution. Similar to Figure 4 but here α-coherence was calculated after subtracting the mean stimulus-evoked response from each trial and pixel (see Materials and Methods). A: α-coherence as a function of time for the collinear (blue), orthogonal (red) and fixation alone (green) conditions averaged over pixels within each of the 4 ROIs (from left to right): V1-CE (n = 555 pixels), V1-flanker (n = 444 pixels), V2-CE (n = 523 pixels) and V2-Flanker (n = 545 pixels). Dashed lines indicate mean±3×SEM. Black horizontal lines indicate upper and lower limits of the α-coherence (mean±3×SEM) in the fixation alone condition. A change in α-coherence is defined by exceeding these limits. There was a significantly higher stimulus-induced α-coherence in the collinear condition compared to the fixation alone condition (Mann-Whitney U-test; p<0.01; averaged 0–100 ms). The inset depicts the pair of ROIs (blue arrow) from which α-coherence was calculated (see Fig. 1E). B: Scatter plots of the average α-coherence (AAC) averaged 0–100 ms after stimulus onset, in the collinear (y-axis) vs. the orthogonal (x-axis) conditions for each pixel in the different ROIs as in A. Most pixels are above the diagonal meaning there is higher AAC in the collinear than the orthogonal condition (Mann-Whitney U-test; p<0.001 for each ROI separately). C: Histograms of time-to-peak (TTP) differences (orthogonal - collinear) of the α-coherence for the all pixel in the four ROIs as in B. Time = 0 indicates that peak α-coherence was reached at the same time window in both conditions. There is significantly lower TTP in the collinear than the orthogonal condition in each ROI (Sign ranked test; p<0.001 for each ROI separately). Data is from 8 recording session and 2 monkeys. (TIF) [file pone.0049391.s003.tif]
